# Salivary miR-31-5p, miR-345-3p, and miR-424-3p Are Reliable Biomarkers in Patients with Oral Squamous Cell Carcinoma

**DOI:** 10.3390/pathogens11020229

**Published:** 2022-02-09

**Authors:** Beáta Scholtz, József Horváth, Ildikó Tar, Csongor Kiss, Ildikó J. Márton

**Affiliations:** 1 Department of Biochemistry and Molecular Biology, Faculty of Medicine, University of Debrecen, 4032 Debrecen, Hungary; 2 Doctoral School of Molecular Cell and Immune Biology, University of Debrecen, 4032 Debrecen, Hungary; horvathjozsef21@gmail.com; 3 Department of Oral Medicine, Faculty of Dentistry, University of Debrecen, 4032 Debrecen, Hungary; tar.ildiko@dental.unideb.hu; 4 Department of Pediatrics, Faculty of Medicine, University of Debrecen, 4032 Debrecen, Hungary; kisscs@med.unideb.hu; 5 Department of Restorative Dentistry, Faculty of Dentistry, University of Debrecen, 4032 Debrecen, Hungary; marton.ildiko@dental.unideb.hu

**Keywords:** oral squamous cell carcinoma, saliva, miR-345, miR-31, miR-424, real-time qPCR

## Abstract

If not detected early, oral squamous cell carcinoma (OSCC) has very poor prognosis, emphasizing the need for reliable early diagnostics. Saliva is considered a promising surrogate biosample for OSCC detection, because it comes into contact with many cells of the tumor mass, providing a comprehensive sampling of tumor-specific biomolecules. Although several protein- and RNA-based salivary biomarkers have been proposed for the detection of OSCC, the results of the studies show large differences. Our goal was to clarify which salivary microRNAs (miRNA) show reliably high expression in the saliva of OSCC patients, to be used as cancer-specific biomarkers, and potentially as early diagnostic biomarkers. Based on a detailed literature search, we selected six miRNAs commonly overexpressed in OSCC, and analyzed their expression in saliva samples of cancer patients and controls by real-time quantitative PCR. Our results suggest that miR-345 and miR-31-5p are consistently upregulated salivary biomarkers for OSCC, and a three-miRNA panel of miR-345, miR-31-5p, and miR-424-3p can distinguish cancer and control patients with high sensitivity.

## 1. Introduction

Saliva has long been considered a prime candidate for OSCC liquid biopsy, because it rinses continuously the oral cavity, effectively sampling the entire surface of the oral mucosa [1,2]. Saliva may collect cells shed by the tumor, as well as different molecules released from tumor cells or by the reprogrammed tumor stroma. Many different salivary protein- and nucleic-acid-based OSCC biomarkers have been tested over the years, as reviewed in [3,4,5,6,7], although the sensitivity of PCR-based detection tilts the balance in favor of RNA- or DNA-based assays. Surprisingly, salivary RNA seems to be relatively intact, presumably due to it being enveloped in extracellular vesicles (EV) such as exosomes, ectosomes, or apoptotic bodies, which have allowed the exploration of the salivary transcriptome in different disease states [8,9]. microRNAs (miRNA) have long been targeted for cancer biomarker development, because miRNA expression profiles of tumors change characteristically, and miRNAs regulate many processes critical for cancer cell survival. The miRNA expression profile specific to OSCC has been studied in detail (see Table 1), but different studies have identified rather different up- or downregulated miRNA sets, possibly due to the well-known heterogeneity of OSCC tumors, although maybe due to varied experimental approaches as well. miRNAs overexpressed by the OSCC tumor mass might be suitable salivary biomarkers [10], but there are several complications. First, miRNAs are selectively released by tumor cells via extracellular vesicles [11,12], and second, saliva is a surrogate sample, containing EVs released by many other normal cells [13,14]. Unfortunately, all this variability has translated into the identification of different miRNA-based OSCC biomarker panels, with little overlap between the studies. Therefore, the aim of our study was to resolve some of these discrepancies, and to identify a reliable salivary miRNA combination for detecting OSCC. We selected six miRNAs to assay their expression in the saliva samples of OSCC patients and controls by real-time quantitative PCR (qPCR): miR-345, miR-424-3p, miR-31-5p, miR-21-5p, miR-191-5p, and miR-184. Based on the literature, these miRNAs all have altered expression in OSCC, in oral precancerous states, or in the saliva of these patients (inclusion criteria), but have no known involvement in oral inflammatory diseases (an exclusion criterion).

Our qPCR analysis suggests that miR-345 and miR-31-5p overexpression can be persistently detected in the saliva samples of OSCC patients, and a three-miRNA panel of miR-345, miR-31-5p, and miR-424-3p can identify OSCC patients with high specificity.

## 2. Results

### 2.1. Selection of Candidate miRNAs as Salivary Biomarkers for OSCC

First, we performed a careful literature search in PubMed to identify miRNAs with frequently altered expression in OSCC, using the keywords “OSCC and miRNA” and “HNSCC and miRNA”, between 1 January 2000 and 31 December 2018. From the resulting list of publications (782), we selected 9 studies that conformed to the following criteria: (1) the initial phase of the studies analyzed more than 20 primary OSCC samples, or saliva of OSCC patients; (2) the initial phase of the studies used a global profiling approach, targeting a large number of miRNAs (qPCR panel, microarray, miRNA sequencing); and (3) after microarray or next-generation sequencing profiling, the results were validated by qPCR in phase II (Table 1, global screening studies, and Supplementary Table). Based on these studies, we identified the following miRNAs with altered expression in OSCC, detected by at least two independent global studies: miR-21, miR-31, miR-424, miR-345, miR-142-3p/5p, miR-146a/b, miR-155, miR-181b, miR-223, and miR-361 (up), and let-7c, miR-99a, miR-125b, miR-133a, and miR-617 (down) (Table 1). Importantly, altered expression of these miRNAs was validated by qPCR analyses at least in the same study, or in independent studies as well (Table 1, targeted qPCR studies, and Supplementary Table). For the final list, we selected only upregulated miRNAs that (a) were supported by independent qPCR validation studies, and (b) were not associated with inflammatory processes in general, or with periodontitis and gingival disease (Table 1, oral inflammatory disease, and Supplementary Table). This resulted in miR-345, miR-424-3p, and miR-31-5p being the putative salivary biomarkers for OSCC. We also added miR-21-5p to the list—although miR-21 is known to have a role in inflammatory processes, it was shown to be overexpressed by many independent studies in OSCC. miR-184 was initially identified as an miRNA overexpressed in squamous cell carcinoma of the tongue, although later studies showed its downregulation in OSCC—to clarify these issues, we decided to include it in the list. miR-191 overexpression was detected only in two studies (one global, one targeted qPCR), although the global study analyzed samples of Hungarian patients, which was of special interest to us. In the end, we selected miR-345, miR-424-3p, miR-31-5p, miR-21-5p, miR-184, and miR-191-5p for qPCR analysis in the saliva samples collected in our multicentric study.

### 2.2. Analysis of Differential miRNA Expression in Saliva Samples of OSCC Patients and Controls

We collected uninduced saliva samples from OSCC patients and controls at four independent clinics in Hungary, as part of an ongoing multicentric study for OSCC. The clinicopathological and demographic data of the patients are summarized in Table 2. These samples were used to quantify the expression of the six selected miRNAs by qPCR: miR-345, miR-424-3p, miR-31-5p, miR-21-5p, miR-184, and miR-191-5p.

**Figure 1:**
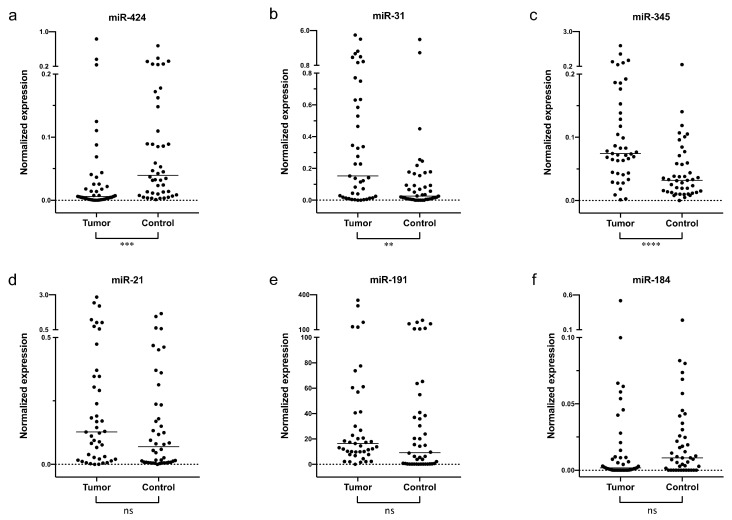
Quantification of miRNAs in the saliva of OSCC and control patients. miRNAs were quantified by RT-qPCR, normalized to the reference gene SNORD60. The figures show the medians for the OSCC (tumor) and control groups, with 43 and 44 samples, respectively. (**a**) miR-424-3p; (**b**) miR-31-5p; (**c**) miR-345; (**d**) miR-21-5p; (**e**) miR-191; (**f**) miR-184. The stars indicate statistically significant differences, as determined by Mann–Whitney U test: **** *p* < 0.0001, *** *p* < 0.001, ** *p* < 0.01. ns, not significant.

**Figure 2:**
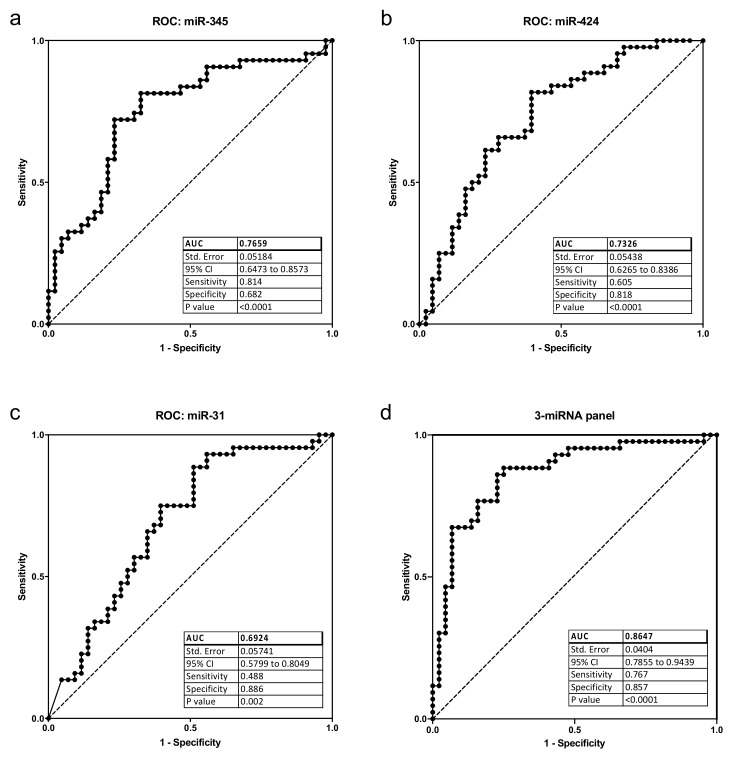
ROC and multiple logistic regression analysis of 3 miRNAs. (**a**) ROC of miR-345; (**b**) ROC of miR-424; (**c**) ROC of miR-31; (**d**) multiple logistic regression analysis with miR-345, miR-424, and miR-31. CI, confidence interval.

Our qPCR analysis showed that miR-191 was present at the highest level in the saliva samples (average C_T_ = 26.8), whereas miR-184 was present at much lower levels, and was undetectable in 11 OSCC and 12 control saliva samples. Normalized salivary miR-31 and miR-345 expressions were higher in OSCC patients, and miR-424 expression was lower in OSCC patients compared with controls (Figure 1a–c). On the other hand, expressions of miR-21, miR-191, and miR-184 were not significantly different between OSCC and control samples (Figure 1d–f), although the generally low level of miR-184 made this assessment less reliable. ROC curve analysis of miR-424, miR-31, and miR-345 showed that each miRNA had a limited power by itself to differentiate between OSCC and control samples, with miR-345 having the largest AUC at 0.77 (Figure 2a–c). However, a combination of the three miRNAs could differentiate well between OSCC and control samples, with AUC at 0.87, specificity at 0.77, and sensitivity at 0.86, as demonstrated by multiple logistic regression analysis (Figure 2d).

## 3. Discussion

Based on their altered expression in OSCC tumors, as determined by independent studies, we selected six miRNAs for qPCR-based quantification in the saliva samples of OSCC patients and controls: miR-345, miR-424-3p, miR-31-5p, miR-21-5p, miR-191-5p, and miR-184. miR-184 was undetectable in too many OSCC and control samples, which makes it unreliable as a biomarker. We demonstrated an overexpression of miR-31 and miR-345, and, surprisingly, a lower expression of miR-424 in samples of OSCC patients, whereas the OSCC-specific upregulation of miR-21 and miR-191 in the saliva samples could not be validated. The relatively small sample size is a limitation of this study, which may bias the evaluation of our results. Despite this, however, we found statistically significant and biologically relevant differences in the expression of three of the selected six miRNAs between OSCC and control saliva samples. Moreover, the three-miRNA panel of miR-345, miR-31-5p, and miR-424-3p could distinguish cancer and control patients with remarkably high specificity and sensitivity, as proven by the ROC analysis.

miR-31 is clearly an oncogenic miRNA, upregulated in different cancer types, which was shown to regulate the stemness transcription factors Nanog/OCT4/Sox2 in cancerous tissue [52,53]. Overexpression of miR-31 in OSCC tumor tissue has been demonstrated repeatedly, and our results corroborate previous observations that this is mirrored in the saliva samples of OSCC patients. A recurring problem in salivary biomarker studies is the confounding effect of inflammation in the oral mucosa, even in tumor-free individuals; however, importantly, miR-31 expression was shown to be suppressed in periodontitis [44]. Other studies have demonstrated miR-31 overexpression in samples of patients with precancerous oral lesions, and in patients with small OSCC tumors as well [54,55,56]; therefore, we suggest that salivary miR-31 might even be reliable as an early biomarker for malignant oral lesions. Our previous work demonstrated that salivary IL-6 is a highly sensitive biomarker of OSCC; thus, we find it significant that both IL-6 and miR-31 are known to contribute to cancer stem cell maintenance in different tumor types [57,58,59,60]. In addition, IL-6 and miR-31 expression are closely co-regulated: IL-6 was shown to activate the Hippo pathway and to upregulate miR-31-5p, resulting in experimental cancer in mice [61]; at the same time, downregulation of miR-31-5p attenuated IL-6 production and release in LPS-induced murine acute lung injury [62].

The role of miR-345 expression in cancer is more variable—the first studies have identified its overexpression in mesothelioma and OSCC [15,17,63], but subsequent studies have suggested a tumor suppressor role in many other cancer types [64,65,66,67,68,69]. Our results show that the previously described overexpression of miR-345 in OSCC tumor tissue is translated into higher salivary expression of this miRNA, differentiating well between OSCC and the control. Other studies have shown an association of miR-345 overexpression with increased levels of dysplasia in oral leukoplakia [30], raising the possibility that miR-345 may also serve as an early diagnostic biomarker for OSCC. Importantly, because miR-345 expression is suppressed in many other cancer types, its overexpression might be a very specific biomarker to OSCC.

The downregulation of miR-424 in OSCC saliva samples came as a surprise, because earlier studies have detected its upregulation in OSCC tissue and in cutaneous squamous cell carcinoma [16,18,19,70], and suggested a role for tumor cell migration and invasion, as well as for sprouting angiogenesis [71,72]. However, miR-424 may act as a tumor suppressor in other cancer types [73,74,75,76], and it also has a complex immunoregulatory role, participating in the regulation of monocyte differentiation, checkpoint control, and the antimicrobial response [77,78,79]. Lower expression of miR-424 in the saliva of OSCC patients suggests an active, systemic suppression of its production or release from the cells of the oral cavity, probably initiated by the tumor itself. Exosomal miRNA loading and release is a regulated process [11,12,80,81], but its regulation by OSCC cells and the possible advantage of systemic miR-424 suppression by the tumor has not yet been explored. miR-21 is overexpressed in many cancer types, including in OSCC; however, similarly to miR-424, it also plays a role in regulating immune processes. The highly conserved miR-191 was also considered to be a promising biomarker, partly because it is overexpressed in many different cancer types, and also because it was found to be overexpressed in OSCC tumors of a Hungarian patient cohort. Although more control patients had very low miR-21 or miR-191 levels in their saliva, the expression differences between the OSCC and control samples were not significant. Some of these discrepancies might be explained by the fact that saliva may collect significant amounts of miRNAs from other areas of the oral mucosa as well. miR-21 is produced during immune cell maturation and in response to pro-inflammatory stimuli [81,82,83,84,85], but its effects appear to be highly context-dependent and different in specific disease states [86,87,88,89]. miR-21 has also been implicated in periodontal disease in recent years, as reviewed in [90], suggesting that this underlying condition of the individuals in our cohort might obscure the difference between cancer patients and controls. The role of miRNAs in oral inflammatory diseases is actively researched, but the salivary miRNA representation of these disease states is not well characterized.

The issue of salivary miR-191 is more complex, despite the negative results of our study. The highest overall salivary expression of miR-191 suggests that it might have some maintenance role in the oral mucosa or salivary glands. Some studies have used miR-191 as an endogenous reference in salivary samples [19,91], but our results captured highly variable expression levels in both OSCC and control samples, warranting caution. Although the sample size was small, seven control patients had exceptionally high miR-191 expression, which was not correlated with a similarly altered expression of the other miRNAs. One possibility to consider is the complex regulation of miR-191 expression by hormones, environmental factors, and dietary components [92,93,94]. miR-191 expression is quite stable in plasma, but the oral mucosa is more exposed to various compounds from food, drinks, tobacco, etc., and what effect the short- or long-term exposure might have on the activity of the miR-191 locus has not been tested. It should also be pointed out that miR-21 and miR-191 expression is altered in other disease types as well, which is reflected in the plasma levels of the patients, and it is currently unclear whether and to what extent these changes are transferred into other biofluids such as saliva.

## 4. Conclusions

In summary, our study identified a three-miRNA panel suitable for the detection of OSCC from saliva, consisting of miR-345, miR-31-5p, and miR-424-3p. Thus far, no single miRNA has been shown to have enough discriminatory power by itself in cancer—a combination of several miRNAs is always required to differentiate cancer patients from healthy subjects. Our results also highlight the difficulties in working with a surrogate sample, saliva, which acquires biomolecules not only from the tumor tissue, but from the oral mucosa and from the salivary glands as well. The majority of salivary miRNAs are probably found within extracellular vesicles, protected from RNases, but the regulation of cellular processes selectively transferring miRNAs into these vesicles is not well understood. This might contribute to the phenomenon that the altered miRNA profile of OSCC tumors is mirrored only in part in saliva. Different studies suggest different miRNA combinations for OSCC detection, and additional work is required to address the biological or technical differences leading to such variability. miR-31-5p and miR-345 overexpression seem to be consistent, and miR-345 upregulation is quite specific to OSCC; therefore, these salivary miRNAs are suggested as constant components of miRNA biomarker panels for OSCC. Lastly, given the shared role of IL-6 and miR-31 in cancer stem cell function, and the OSCC-specific increase in salivary IL-6 mRNA, adding IL-6 mRNA to the miRNA biomarker panel may improve both the sensitivity and specificity of OSCC detection.

## 5. Materials and Methods

### 5.1. Study Subjects and Salivary Sample Collection

The study was approved by the Institutional Review Board of the University of Debrecen (No. 3244-8/2011, No. 3722-2012) and by the Scientific and Research Ethics Committee, Medical Research Council, Hungary [(693/PI/12.) 45038-1/2012/EKU]. Sample collection was performed in accordance with The Code of Ethics of the World Medical Association and the ethical standards of the 2000 Revision of the Helsinki Declaration. All participating subjects provided signed informed consent. OSCC patients (43) and controls (44) were participants of our previous multicentric study, and their clinicopathological and demographic data are summarized in Table 2. DMFT is the sum of the number of decayed, missing due to caries, and filled teeth in permanent dentures. The gingival index (GI) scores the inflammation of the gingiva characterized by edema, redness, swelling, and spontaneous bleeding on a 0–3 scale. Ethanol consumption and smoking habits were characterized as described previously [95].

### 5.2. Saliva Processing, RNA Isolation, Reverse Transcription, and Real-Time Quantitative PCR

Salivary sample collection, pre-processing, and total RNA isolation were carried out as described before [95]. Successful isolation of the small and large RNA fraction was verified with Agilent 2100 BioAnalyzer, using the RNA 6000 Nanokit (Cat. No. 5067-1511, Agilent, Santa Clara, CA, USA). Based on the Agilent analysis, 87 samples (43 OSCC and 44 control) were selected for miRNA quantification. Subsequently, 3 µL of each saliva total RNA sample was reverse-transcribed (RT) using the TaqMan™ MicroRNA Reverse Transcription kit (Cat. No. 4366596, Thermo Fisher Scientific, Applied Biosystems^®^, Waltham, MA, USA), according to the manufacturer’s instructions, in a total volume of 15 µL, using a premixed primer pool. Equal volumes of the RT primers for each miRNA and random hexamer primers for the reference gene, SNORD60, were pooled, and 9 µL of the pooled primer mix was added to the RT reaction of each sample. Expression of the miRNAs and SNORD60 was quantified using real-time quantitative PCR with TaqMan^®^ assays on a QuantStudio™ 12K Flex Real Time PCR System (Applied Biosystem^®^), with the same reaction conditions as before, using cDNA samples diluted 5-fold as templates [95]. TaqMan™ qPCR assay IDs were as follows: hsa-miR-345 ID:002186; hsa-miR-424-3p ID:002309; hsa-miR-184 ID:000485; hsa-miR-21-5p ID:000397; hsa-miR-31-5p ID:002279; hsa-miR-191-5p ID:002299; and SNORD60 ID:Hs03309311_s1. The qPCR assays for miRNAs came with the miRNA-specific RT primers. SNORD60 was selected as the reference gene because it is present in whole saliva [96], and neither its host lncRNA gene, SNHG19, nor SNORD60 itself, was reported to have altered expression in OSCC.

### 5.3. Data Processing and Statistics

Raw data analysis was performed using the ExpressionSuite Software (version 1.0.3 for Microsoft^®^ Windows^®^, Thermo Fisher Scientific, Waltham, MA, USA), and normalized gene expression was calculated with the ddC_T_ method, with the 2^−dCT^ values presented in the graphs [97]. Expression of the reference gene, SNORD60, was quantifiable in all samples, with similar amplification curve profiles and with an average raw C_T_ = 30.3, indicating that RT was successful and PCR amplification was not inhibited in the samples. Quantification limits of the miRNA qPCR assays were C_T_ = 38. Samples with Ct > 38 were defined as non-quantifiable positive, and their C_T_ values were uniformly set to 39. Differential expression of the miRNAs was assessed between the patient cohorts with nonparametric Mann–Whitney U tests on normalized expression values. Receiver operating characteristic (ROC) curve analysis and multiple logistic regression analysis was carried out on the same datasets to evaluate the discriminative power of the miRNAs, and the areas under the curve (AUC) were determined. All statistical analyses were performed with GraphPad Prism (version 9 for Mac, GraphPad Software, San Diego, CA, USA, www.graphpad.com, accessed on 8 March 2019).

## Figures and Tables

**Table 1:** Selection of miRNAs as candidate biomarkers for OSCC. The table shows the 19 miRNAs with altered expression in OSCC, supported by multiple studies. For each OSCC-specific miRNA, the table indicates the publications identifying their altered expression (a) by global screening approaches, (b) by targeted qPCR studies, and (c) the publications showing the connection between inflammatory oral diseases and miRNAs. The 6 miRNAs making the final list are in bold.

Upregulated miRNAs	Global Screening Studies	Targeted qPCR Studies	Oral Inflammatory Disease
**miR-345**	[15,16]	[17]	
**miR-424**	[16,18]	[19]	
**miR-31**	[16,20]	[19,21,22]	
**miR-21**	[15,16,18,20,23,24,25,26]	[2,17,27,28,29,30,31,32,33]	
**miR-184**	[15,34]	[2,27,35]	
**miR-191**	[24]	[36]	
miR-142-3p	[18,23]	[1,19]	[37,38,39]
miR-142-5p	[18,23]	[1,19]	[37,38,39]
miR-146a	[15,16]		[37]
miR-146b-5p	[16,23]	[40,41]	[37]
miR-155	[16,24]	[42,43]	[37,44]
miR-181b	[15,16]	[17,45]	[46]
miR-223	[16,20]	[19,47]	[37,39,44]
miR-361-3p	[18,23]		
**Downregulated miRNAs**			
let-7c	[18,20,25]		
miR-99a	[16,18,25]	[47,48]	
miR-125b	[16,18,25,26]	[17,49,50,51]	[44]
miR-133a	[20,23]		
miR-617	[16,18]		

**Table 2:** Clinical and demographic characteristics of patients and controls. DMFT, decayed, missing, and filled teeth index. GI, gingival index. N, number of individuals. NA, data not available.

		Patients (N = 43)	Controls (N = 44)
Sex (N (%))	Male	28 (65%)	16 (36%)
	Female	15 (35%)	28 (64%)
Age (mean, years)		57.9	57.6
Histological grade (N (%))	G1	8 (18%)	-
	G2	18 (42%)	-
	G3	9 (21%)	-
	NA	8 (19%)	-
Stage (N (%))	St I	9 (21%)	-
	St II	14 (32%)	-
	St III	5 (12%)	-
	ST IV	8 (19%)	-
	NA	7 (16%)	-
DMFT (mean (N))		25.35 (35)	24.73 (37)
	NA	- (8)	- (7)
GI (mean (N))		0.3 (18)	0.5 (35)
	NA	- (25)	- (9)
Ethanol consumption (N (%))	on weekly basis	12 (28%)	4 (9%)
	rarely or never	23 (53%)	23 (52%)
	NA	8 (9%)	17 (39%)
Smoking (N (%))	regular	24 (56%)	6 (14%)
	occasional/non	12 (28%)	31 (70%)
	NA	7 (16%)	7 (16%)

## Data Availability

The data presented in this study are available on request from the corresponding author.

## Supplementary Materials

The following supporting information can be downloaded at: https://www.mdpi.com/article/10.3390/pathogens11020229/s1, Table S1: Supplementary Table, OSCC miRNA screening studies.
